# “Here comes Bio-me”: An analysis of a biobank campaign targeted at children

**DOI:** 10.1177/09636625211022648

**Published:** 2021-06-19

**Authors:** Karoliina Snell, Heta Tarkkala

**Affiliations:** University of Helsinki, Finland

**Keywords:** biobanking, children, genes, metaphors, science education

## Abstract

Finnish biobanks have started to recruit children. The national supervising authority has emphasized the centrality of providing children with age-appropriate information. We analyzed one such campaign. We argue that by simplifying the complex socio-technical arrangements of biobanking with the introduction of a new metaphor-like concept, “Bio-me,” the campaign presents a misleading and reductionist picture of data-driven biomedicine and biobank participation. First, the Bio-me character seems to bear similarities to the seventeenth-century explanations of embryological development. Second, the focus in the campaign is on biological material while crucial connections to different sorts of data are ignored. Third, we point to the absence of verbal references to genes and DNA, although the prevailing visualization comprises the double helix. We argue that the campaign has potential to contribute to public misunderstanding of science by introducing a new term that has little connection to actual biology or scientific practices it tries to promote.

## 1. Introduction—Finnish biobanking and a campaign for recruiting children

Biobanks are research infrastructures where biological material from humans (such as blood, tissue, and saliva) and related data (such as laboratory results and diagnostic information) are stored for future research purposes. Biobanking operations in Finland were officially established in 2013 when a law on biobanks (Biobank Act 688/2012) was enforced. All biobanks in Finland have to be registered in a national register that was supervised from 2013 to 2019 by Valvira (the National Supervisory Authority for Welfare and Health). Currently, the Finnish Medicines Agency Fimea, which supervises and develops the pharmaceutical sector in Finland, maintains the register. At present, there are eleven registered biobanks in Finland: six clinical biobanks connected to hospital districts, the biobank of the National Institute of Health and Welfare, the biobank of the Finnish Blood Service, one disease-specific biobank, one governed by the University of Oulu, and one operated by a private healthcare company. During the initial years of biobanking, several old diagnostic collections were transferred to the biobanks, and collection of new samples was started (Salokannel et al., 2020; Tarkkala, 2019). At first, the target was the adult population, but in 2016, some biobanks started to collect samples from children. The recruitment of children started at the Helsinki Biobank and other clinical biobanks have since followed. Despite active international discussion about concerns related to children as biobank donors, and whether they should be re-contacted once they are adults (e.g. Giesbertz et al., 2016; Gurwitz et al., 2009; Hens et al., 2011), there has been very little public discussion in Finland about the ethical implications of collecting children’s samples in biobanks.

In 2016, however, the supervising authority Valvira issued a letter of guidance for biobanks containing guidelines concerning children (Valvira, 2016). According to the Biobank Act, the collection of new samples requires informed consent from the participant. Since the Biobank Act did not deal with sample and data collection from children in detail, Valvira’s letter of guidance sought to address this issue. The guidelines state that a guardian should sign the consent form on behalf of a minor, but that the consent should reflect the presumed will of the minor. If the child can understand—in accordance with her or his development stage—the meaning and nature of biobank research, written consent from the minor should be acquired as well. As most Finnish adults at that time were unfamiliar with biobanks (Snell, 2017; Snell and Tarkkala, 2019), it was natural to presume that children would also need to be educated on the matter in order to make an informed decision. Valvira emphasized that prior to consent being given, biobanks need to inform children about “the nature of biobanking, possible harms, that consenting is voluntary and other issues regulated in the Biobank Act § 4. The information regarding biobanking should be given in the most clear and understandable way possible” (Valvira, 2016). Information given to the participant should include a description of the nature of biobanking, including the use of genetic and other data, and the possible need to combine data from different registers.

Valvira also advised biobanks to use supportive material (such as mobile apps, games, posters, or websites) for disseminating the information to children and stressed that it would need to be tailored to the appropriate age level. Some biobanks responded to this quickly and Auria Biobank, for example, introduced a mobile game targeted at teenagers and young adults, while Helsinki Biobank commissioned a campaign from a consulting company to address this need. Helsinki Biobank’s campaign “Here comes Bio-me” included a video and a leaflet and was launched in 2018. The material has been partly financed by the national network of biobanks BBMRI.fi and is therefore utilized by other biobanks as well.

This article presents a content analysis of the “Here comes Bio-me” campaign material and the epistemic claims made in the campaign. Our focus is especially on the metaphors used and created for the campaign, and how the campaign uses these metaphors to depict what biobanks do and what is stored in them, and how this relates to the child as biobank participant. This is more or less the focus of the information campaign itself as well.

We examine the campaign using visual and textual analysis, focusing in particular on its main character—“Bio-me”—which we view as a metaphor. In this article, we approach metaphors as vital tools of science communication, where they function as a means of furthering understanding of complex issues (Väliverronen, 1998; Väliverronen and Hellsten, 2002). Metaphors are, to put it simply, about “understanding and experiencing one kind of thing in terms of another” (Lakoff and Johnson, 1980: 5), and are able to evoke associations and to act as a bridge between the known and the unknown (Yu, 2017). Metaphors have also a connective function, and they may be used to “mobilize thinking and action in certain directions by evoking feelings and establishing links with already existing norms” (Väliverronen, 1998: 22). This way metaphors are “generative metaphors” (Schön, 1993)—frames that shape thinking and can help to connect one’s own actions with scientific practice. The creation of an imaginary about how children participate in biobanking in the form of Bio-me character connects it as a metaphor to efforts to convince and persuade (e.g. Schön, 1993).

Metaphors are often used in science communication and in illustrating the possibilities and methods of new technologies such as genetics (Ceccarelli, 2004; Hellsten, 2000; Keller, 1995; Nelkin and Lindee, 1995; Nerlich et al., 2002; Nerlich and Halliday, 2007; Nerlich and Hellsten, 2004; Saukko, 2017). Metaphors such as “the book of life,” “the blueprint” (Condit, 1999; Nelkin and Lindee, 1995), or “genetic code” (Knudsen, 2005) are some of the most well-known and researched metaphors used in connection with genetics. Unlike these metaphors that are widely used in media and public communication, Bio-me is a metaphor created for this particular campaign. Thus, our case does not come from mass media, nor is it descriptive of the yet unknown object of science (see, for example, Bostanci, 2010: 468–469).

Most of the studies examining metaphors have focused on verbal metaphors, while our focus is on both verbal and visual metaphors and their relation to epistemological claims—how they represent what they are supposed to represent. Yu (2017) claims that visual metaphors basically work in a similar way to verbal metaphors by connecting two concepts, frequently the known and the unknown, as in science communication. Images can serve as portals or gates in public communication to introduce new scientific and technical advancements (Gigante, 2018). Popular visual communication of science has a long history, ranging from data visualizations to artistic illustrations, drawings, and photographs (Gigante, 2018; Krause, 2017). Moreover, visual metaphors can take many forms (El Refaie, 2003), but are often understood as consisting of a visual rhetorical figure that connects two images. Phillips and McQuarrie (2004) differentiate between three ways of structuring such images: juxtaposition (two images side by side), fusion (images merged together), and replacement (an image inserted in the place of another). In addition, they point to different meaning operations—or the kind of cognitive processing that is required to comprehend the picture—such as understanding connections, similarity, or opposition. The relationship between different visual elements can also be understood in terms of the signifier and the signified—referring to what is pictured and what it represents (El Refaie, 2003; Greenwood et al., 2019).

While metaphors can be productive and help to make complex issues accessible (Ratto, 2006), their use has also been criticized. The genetic blueprint metaphor, for example, has been criticized for being determinist and for oversimplifying complex phenomena (Nelkin and Lindee, 1995; Rothman, 2001). It has been pointed out that metaphors can contribute to public misunderstanding and hence they need to keep pace with recent advances in scientific understanding (Taylor and Dewsbury, 2018). Our research contributes to these discussions on the potential for causing misunderstanding or miscommunication (e.g. Hellsten and Nerlich, 2008; Morgenstern et al., 2015) by addressing contradictions in metaphors used in science communication. We investigate what the campaign under study implies, and what its main visual character represents in relation to what it is supposed to be informative of. Our aim is to discuss the metaphor-like character of Bio-me in relation to biobanks and their samples and, in so doing, to demonstrate the potential for misunderstanding related to biomedical research and the role that biobanks play in this. The analysis addresses what remains unsaid in the campaign, on one hand, and discusses what the chosen foregrounded information seems to imply, on the other hand.

## 2. Material and methods

The research focuses on the “Here comes Bio-me” (*Täältä tulee Biominä*) campaign materials targeted at children, which comprise an animated video and a leaflet. Coloring books and activity books have also subsequently been produced. The campaign was launched for the purpose of educating and informing minors about what biobanks are, what they do, and what biobank sampling means. The video has been shared on the Internet and the Facebook pages of several biobanks and the BBMRI.fi network of Finnish biobanks and is openly available on YouTube. There are two versions of the video, a shorter (2 minutes 28 seconds) and a longer edition (3 minutes 39 seconds). The content is the same in both, but the longer one also includes a description of how biobanks are governed and what kind of people or professionals work there. Our analysis is concerned with the shorter video only as our focus is on the sample and the role of the child as a biobank participant, as this is where we identify the greatest potential for misunderstanding.

The leaflet follows the same storyline and contains more or less the same information as the video. The narration in the video is more condensed in places, however. Both of the materials are in Finnish and the translations used here are our own.

Our interest in analyzing the campaign material was prompted by the main figure and its name—“Bio-me,” which we felt needed clarifying. It became apparent to us that the figure is not a scientific or an established concept. Instead, it has been developed specifically for this campaign and has metaphor-like characteristics, and thus we were intrigued to analyze it as a metaphor. We analyzed the video and leaflet by identifying other metaphors as well as connections, similarities, contradictions, and oppositions produced inside the campaign (Phillips and McQuarrie, 2004) and in relation to their context in the outside world. Metaphors were examined as elements of communication and epistemological claims of the campaign. The methodological approach represents a content analysis that combines textual and visual analysis (Harrison, 2002; Yu, 2017). Hence, we focused both on the narrated and the written text as well as the visualizations in the material and asked: What is this and what does it represent and claim? How does the text, visualizations, and the metaphors in them correspond to contemporary biobanking practices? Thus, we identified more explicit links with the external surroundings, culture, and knowledge that go beyond the superficial information provided by the text or image itself (Greenwood et al., 2018) and analyzed what is left out of discussion as a result of the chosen narratives, visualizations, and metaphors. As Väliverronen (1998) has pointed out, a metaphor “has dual character: it may reveal something new, but also hide something else” (pp. 19–20). This dual character of metaphors is integral part of the Bio-me campaign.

Over the years, we have worked on several projects that analyze biobanks and their publics, as well as the technical and scientific practices adopted by biobanks (see Cañada et al., 2015; Snell, 2019; Snell et al., 2012; Snell and Tarkkala, 2019; Tarkkala, 2019). We have participated in several public seminars and events on biobanking and followed the development of the field in Finland since the beginning of the 2010s. While we do not utilize research materials from these projects, the understanding we have gained while engaged in the projects, coupled with our background knowledge of Finnish and European biobanking, informs the analysis and has guided us to direct our gaze toward this particular public communication campaign.

## 3. Preformation and construction of a Bio-me

The key illustration in both the video and the leaflet is that of the Bio-me figure. According to Gigante (2018), the figure can be thought of as a portal image that serves as an entry point into scientific discourse or can be conceived of as an introduction to a subject. Cover images and visualizations appearing before the related text are examples of portal images. The video begins with the Bio-me figure and the image is also on the cover of the leaflet.

**Figure 1.**
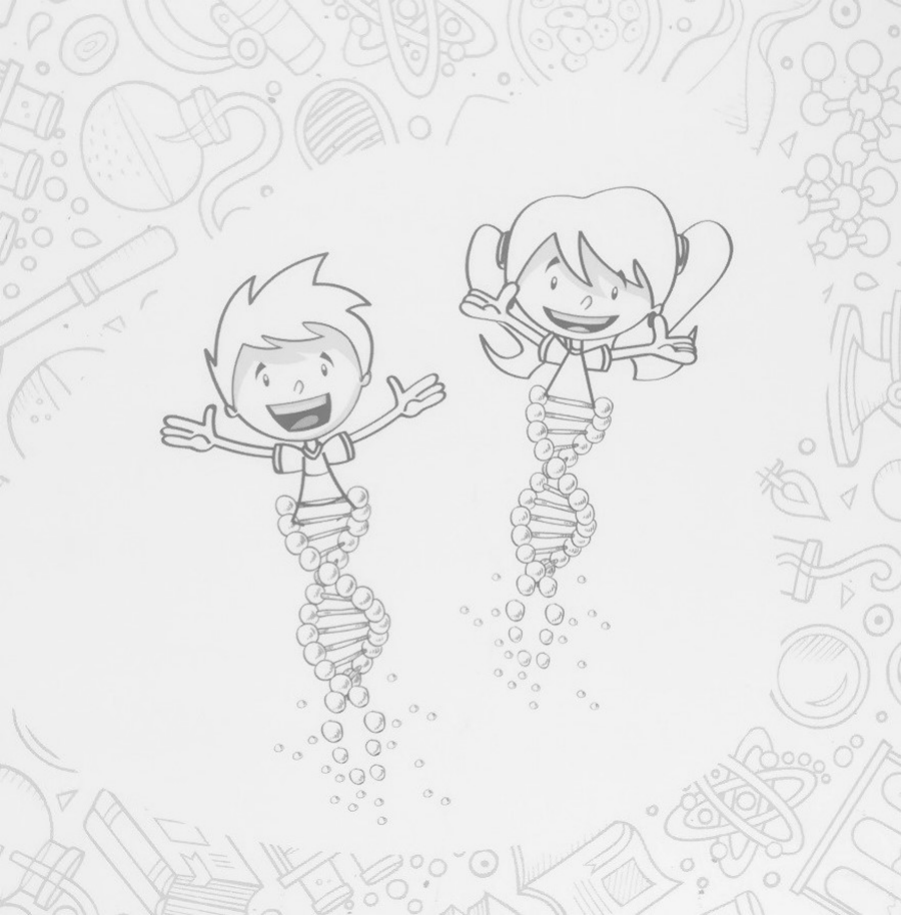
Bio-me characters as pictured on the back cover of the leaflet. Material published by Helsinki Biobank and BBMRI.fi.

Like many visual metaphors, the child-like Bio-me (see Figure 1) consists of two components: double helix and a child. The double helix spiral functions as the lower part of character’s body. The other component is a large smiling head of a child as well as his or her upper body and outstretched arms. This combination of child and double helix in Bio-me can also be considered as a fusion metaphor (Phillips and McQuarrie, 2004; Yu, 2017). There are both female and male versions of Bio-me—the boy drawn with spikey hair and the girl with fluffy pigtails; both children are white and attached to similar double helixes. The boy features on the cover of the leaflet and inside it, and plays a major role in the video, while the girl appears inside the leaflet, on the back cover, and in the concluding scene in the video. The whiteness of the Bio-me connects with the representation of the homogeneous population Finnish biobanks claim they store. Genetic homogeneity of the population is utilized as a marketing strategy for attracting international research and business as it is argued to offer unique possibilities to do biomedical research efficiently (Tarkkala, 2019; Tarkkala and Tupasela, 2018; Tupasela, 2017).

The video starts with the title “Here comes Bio-me” and the Bio-me figure. This is followed by a narration about how scientists are trying to help people all over the world by finding new cures for diseases, and that “you have the opportunity to help them.” It is followed by sentences explaining how every person is a bit different because of the “building material inside us” that determines “what we look like, what we like, and even what diseases we have a tendency to get.” The video then introduces Bio-me. A happy animated boy appears in the center of the screen, and attention turns to the boy’s left hand. A magnifying glass appears and focuses on his hand, where a Bio-me figure pops up. The figure has the same face as the boy. The female narrator says, “Bio-me is a microscopic small version of you or of any other child.” The text Bio-me appears next to the magnifying glass and an arrow connects the text to the figure.

**Figure 2.**
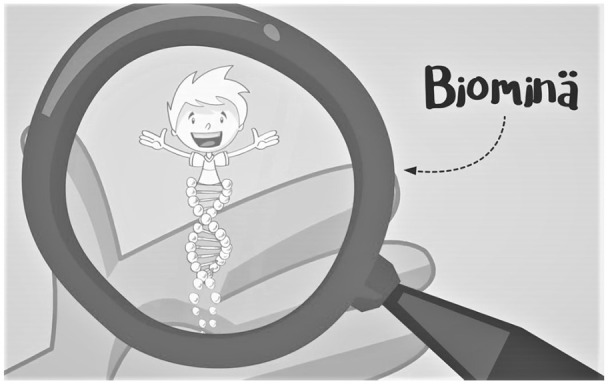
Bio-me figure through a magnifying glass in the video. Material published by Helsinki Biobank and BBMRI.fi.

The figure of Bio-me points both verbally and visually to the similarities between the boy and his Bio-me, which the narration describes to be a smaller, microscopic version of the boy. The magnifying glass demonstrates this visually (Figure 2). Verbally, both the content of the text and the suffix “me” draw attention to the similarities. Thus, the character represents a version of the child—with some sort of shared features existing in both the child and the Bio-me. The Bio-me also becomes connected to the preceding narrative on the building material inside us, not least since it had apparently been in the boy’s hand the whole time.

**Figure 3.**
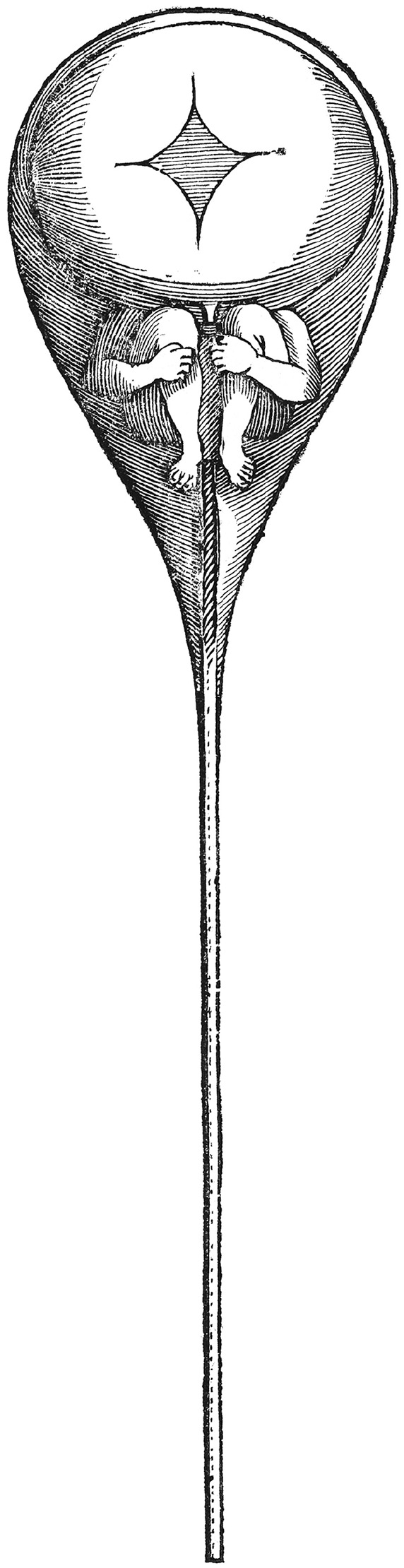
A homunculus inside a human sperm cell. Source: N. Hartsoeker, Essay de dioptrique. Credit: Wellcome Collection. Attribution 4.0 International (CC BY 4.0).

This figure, and its presentation, resembles a “homunculus” or the “little person” imagined in the seventeenth and eighteenth centuries to be inside sperm cells—not only as a visual metaphor, but also in terms of ontology. In 1694, Nicolaas Hartsoeker, a Dutch mathematician and scientist, claimed to see a tiny figure of a man inside a sperm cell when examining them under a microscope. His drawing of this tiny human inside the head of the sperm cell (Figure 3) became an iconic figure that crystallized a contemporary theory in embryology known as preformationism (Correia, 1998; Lawrence, 2008). Preformationism was a theory of embryological development that was grounded in the idea that humans (and animals) develop from miniscule versions of themselves. These little persons have all the same parts as grown humans, albeit on a much smaller scale. Just like a homunculus, the Bio-me is depicted as a smaller biological version of the original person.

We do not want to imply that children watching the video or reading the leaflet are familiar with the homunculus concept, but seek to emphasize the ontological similarities between the Bio-me metaphor and this three-century-old concept. Bio-me comprises a similar predetermined idea of an entity. As a metaphor, Bio-me points both visually and as narrated to the similarities between the smaller and the larger versions of the child and suggests predetermination of some kind—from predispositions to illness to the way we look. In the video, the figure emerges visually through a magnifying glass centered on the child’s hand, and in the leaflet it is stated that: “All of us have an individual Bio-me. Mom, Dad, you . . . and me.” In this sense, Bio-me refers not only to the existence of this entity called Bio-me but also to shared biology; all humans have a Bio-me that seems to be one, coherent, small copy of the human it originates from. The term Bio-me has biological implications as well. Human biology is shared and natural, but also individual and personal.

In contrast to the homunculus, Bio-me does not refer to the development from a small human being into a full-grown one, but to miniscule components or building materials that are inside one’s body. The location of Bio-me inside the child is, however, not particularly clear. While there were two competing strands of preformationism—some thought that the homunculus was located in egg cells while others placed it in the sperm cell—the location of Bio-me is similarly imprecise. It is explained that a sample is often taken from blood but that there are other ways of sample-taking. It remains for the reader or viewer to contemplate exactly where Bio-me is located.

However, later on in the video and also on the second page of the leaflet, it is pointed out that Bio-me needs to be constructed. The Bio-me concept and its visual portal image type of utilization depict a predetermined and predefined vision of an entity defining our heredity, development, and susceptibility to disease, but this is contradicted by the construction metaphor. The leaflet states that: “Now you can help too. A Bio-me can be made out of you with the help of the biobank . . . Perhaps you will also have your very own Bio-me that lives in a biobank at some point”.

Now there are two mixed metaphors (Ceccarelli, 2004) or contradictive ways of representing the object stored in a biobank. The construction metaphor switches things around by presenting Bio-me not as a predetermined biological entity, but in opposition as something that can be assembled from a person. It can then start to live a life of its own, which is another intriguing metaphor that enhances the conception of Bio-me as a biological entity. This contradiction between a pre-existing Bio-me and the need to construct it is present throughout the material and, as Gigante (2018) has pointed out, portal images can yield conflicting interpretations as a text progresses. The script continues by stating that prior to the construction of one’s Bio-me, a consent form must be signed. The consent “gives a biobank your permission to store and examine your Bio-me,” but there is no mention of giving permission to construct the Bio-me. So what, then, is a Bio-me? And what exactly is constructed or stored and examined in a biobank that is considered to be such an entity?

## 4. Biology and sample centeredness

The purpose of biobanks is to store samples of human origin together with associated health data (e.g. electronic health records, laboratory results, health surveys) to be used in future scientific research and development. Increasingly, samples are analyzed and processed as digital data, and hence researchers might not see the so-called wet samples at all. Furthermore, the associated health data can contain information based on other samples taken from the individual for diagnostic purposes. Consequently, the line between biobank samples and data is blurred, but researchers and biobank managers generally acknowledge that a sample is worthless without any associated data (Tarkkala, 2019: 43–44), and storing samples is more expensive than storing data. The Bio-me campaign, however, focuses only on biological samples and bypasses the idea of a “digital-me” as well as the significant role that data and data accumulation play in furthering biobank research.

In this way, the Bio-me metaphor both reflects and distorts the complex questions of what biobanks store, what a biobank sample is, and how it could be understood. Tarkkala (2019: 48) has argued that, ultimately, a biobank sample is best understood as a data package that is different each time it is enacted and put to use in a research setting. There is no usable and utilizable sample or biological entity as such stored in a biobank. Instead, a biobank houses a sample that can be linked with different sorts of data in different ways according to the research in question. In this sense, a deposit in a biobank is not about the potential of a few test tubes of blood being circulated for different purposes, but about the multiple, potential data links the samples can be enacted with in a specific research setting (Tarkkala, 2019). Thus, the construction metaphor presented in the campaign does reflect the practices associated with biobank research, but omits the multiplicity involved in the actual construction of research data. It also implies that the sample is constructed only once. This omits not only data as such, but also its accumulating nature. Over time, the potential data that can be attached to a sample grows through entries in different registers and via healthcare visits that are registered in centralized electronic patient files. As a result, there is no frozen moment of samples and attached data, but rather the continuous potential for data accumulation and the enrichment of what the biobank stores or constructs over the life course of an individual. This is particularly relevant in the case of children and one of the rationales behind starting to collect samples from children in the first place.

According to the Bio-me material, biobanks construct the Bio-me. However, this construction metaphor concerns only sample processing—for example, the route taken by blood from the body through the lab to the liquid nitrogen freezer—not data. During this process, according to the material, some parts (plasma, red and white blood cells) are removed from the sample and placed in smaller ampules. Nothing except a code is added and the video states: “After this, your sample is concealed in a form whereby no one has a possibility to identify it.” Then the Bio-me figure with the boy’s head and torso is moved to a nitrogen freezer where “your Bio-me sample is waiting for potential research use.” Interestingly, the boy’s face attached to the figure is very recognizable in this process.

But can a biological sample or a sample with an added data package be a microscopic version of you? In the social science literature, there is much discussion of how an individual is represented through their data with concepts such as a digital dossier (Solove, 2004), a digital archive (Waldby, 1997), and a data double (Haggerty and Ericson, 2000; Ruckenstein, 2014). A data double, for example, is a concept developed in relation to surveillance, referring to an abstraction of information in separate flows that can be reassembled for different purposes and that forms a new decorporealized body in the setting in which it is being utilized (Haggerty and Ericson, 2000: 605, 611). Building on expanded uses of this concept, the biobank data, like data doubles, are not “accurate or inaccurate portrayals of real individuals, they are a form of pragmatics”—tied to their utility for given purposes (Haggerty and Ericson, 2000: 614). It is the reassembly of different data sources and the context specificity of the application of data that the Bio-me character obscures. Whereas Ruckenstein (2014) has looked at data doubles in self-tracking—forms of personal data doubles that can be used by individuals themselves—Bio-Me points in another direction, where there is no self-enactment of the data.

## 5. The presence and absence of genes

The video ends with the statement: “Now you know more about Bio-me and the operations of a biobank.” The Bio-me figure is a character and a concept developed exclusively for the purpose of this particular campaign. We have not asked those involved in designing the campaign why they chose to create a new concept that, as we have shown, does not correspond either to biology or to actual practices of technoscience in biobanks. The figure is an assemblage, a combination of a child and a double helix, which points strongly to DNA and genes. The double helix is a well-recognized symbol used on TV, in movies, in advertising, on book covers and so forth to suggest genetics (Yu, 2017). While the idea of genetics is present visually throughout the campaign material, and the double helix is part of the portal image of Bio-me, both the video and the leaflet lack direct references to genes or genetics. Instead, there are loose references to “the building material inside us.”

The leaflet includes only a small “Did you know?” box containing a different type of drawing of a double helix—colorful, and without the bubbles. The text says: “All Bio-mes, like people in general, are a little bit different because of a substance called DNA. DNA determines what we look like, what we like, and what diseases we have a tendency to get.” It continues in even smaller print: “DNA is made up of two connected tracts, which make it look like a ladder.” This mention, not present in the video, is the only textual indication of DNA, while genes as a term are not mentioned in the video or the leaflet at all. Thus, the Bio-me figure introduces the idea of genetics and DNA visually in a campaign that lacks textual references to genes.

In Finland, genes and heredity are a part of the school curriculum for ninth graders (14–16 year olds), but the idea of genes and DNA is familiar to younger children as well. There is little research on how children understand genetics or become familiar with the subject, but a small study indicates that Finnish 11- to 15-year-old pupils have knowledge about genetics and are familiar with the concept of DNA before it is officially taught in school (Tiainen, 2019). Similarly, in a study conducted in Australia, the majority of children between 10 and 12 had some knowledge about DNA or genes (Donovan and Venville, 2014). In an article reporting on a study among 7–10 year olds in the United States, Driessnack and Gallo (2013) state, “In short, children know a lot more than we think they do, at least about genetics.” (p. 178) Mass media and entertainment contribute to this awareness as TV shows and games popular with children are loaded with references to genes, genetics, and DNA (Donovan and Venville, 2014).

## 6. Discussion

We have analyzed the “Here comes Bio-me” campaign that aims to educate Finnish children about biobanks and duly promote their participation in them. We approached this case by considering both visual and textual material and focusing on the metaphors presented in the campaign. Our main focus was the portal image of Bio-me (Gigante, 2018), but we also identified other metaphors, most importantly, the construction metaphor. The campaign responds to the supervising authority’s call to provide supportive material for minors (Valvira, 2016). However, as we have demonstrated, it does not meet Valvira’s conception of the kind of content that should be communicated in an age-appropriate and informative manner. Our main argument is that instead of providing accurate information that supports public understanding of science, the campaign has many characteristics that can contribute to public *mis*understanding of science about what biobanking is as a practice. With the invention of a new metaphor-like figure and Bio-me concept, the campaign detaches biobanking from genetics and health data and associates it with biological predetermination and internal coherence and stability.

According to Valvira, much of the communication for minors should be clarified with the help of supportive material, unlike for adults, for whom informed consent is the main channel of communication (Morgenstern et al., 2015). Among other things, the supportive material should address the use of genetic and health data in biobanking and the linking of samples to register data. Our methodology in this study, combining visual and textual analysis, provides insights into contradictions in the Bio-me campaign, which can cause misunderstanding. There are visible hints about DNA in the main figure but an evident absence of textual references to genetics. Choosing to bypass genetics in explicit terms not only overlooks a major function of biobanking, but also misses an opportunity to educate children about proper science-derived concepts. While more research needs to be conducted on how children understand and become familiar with genetics and DNA, genes are highly visible in the media and in movies targeted at children and young adults and have become part of the public culture (Bates, 2005). As a result, talking about DNA and genetics could resonate more strongly with children than a totally new concept.

In addition to genetics, the campaign lacks references to different types of health data, which are the key component of biobanking. Biobanks, in turn, are an integral part of data-driven medicine, which is vested with political, economic, and scientific expectations. “Here comes Bio-me” does not take data-driven medicine into account, shifting the attention to biology instead and reducing the potential of biobanking to samples. In contrast to much of contemporary social science as well as biomedical discussions, which recognize the increasing importance of various types of health-related data in biomedicine and of developing new treatments (e.g. Prainsack, 2017; Ratto, 2006; Tarkkala, 2019; Topol, 2012), this campaign offers a more traditional version of biomedicine where biology and samples provide solutions by themselves. So traditional, in fact, that it reminded us of a homunculus and preformationism.

Another contradiction that emerged in the campaign is the reference to a predetermined biological entity called Bio-me juxtaposed with the need to construct it in a biobank. Thus, Bio-me is presented as a whole—a microscopic small version of a human inside or on one’s body, and as something that is processed from one’s sample and formed into a whole. Whether two competing metaphors create a venue for understanding or misunderstanding is open to question. Ceccarelli (2004) has argued that if two metaphors used in the same text are complementary to each other or offer a competing interpretation, this can lead to a more comprehensive understanding of difficult subject matter. However, she goes on to say that, in fact, interpreting and using metaphors is usually more complicated than this and it is difficult to foresee how metaphors are interpreted and redistributed. The audience might interpret the metaphors in ways that are not anticipated (Condit, 1999). The interpretation the audience does, or the ways science as practice or knowledge changes over time, and how the metaphor then holds, is not in the hands of the ones who come up with metaphors (Condit, 1999; Copland, 2005; Nelkin, 2004; Taylor and Dewsbury, 2018). Using metaphors to promote biomedicine or educate people is often necessary but difficult, as they entangle epistemology and rhetoric in ways that are sometimes difficult to anticipate, control, or sustain. But the choice of a metaphor might have long lasting consequences in public communication. In the name of responsible science, Larson (2011) calls for more consideration when choosing metaphors and even for testing them with different audiences.

The case presented in this article points to the problems involved in providing information of an age-appropriate level. This is hardly surprising, as questions about the right age for consenting children and when should children be regarded autonomous subjects are ethically and legally disputed issues (e.g. Hens et al., 2011). The age range of the target audience of the Bio-me campaign is unspecified, but a campaign for children of any age should be both informative and interesting enough to capture their attention toward the topic, with the child eventually reaching some kind of state of being “informed” (Barr, 2006). Even though there is a delicate balance between offering too little overly simplified information that contains misrepresentations (Condit, 2007) and too much overly complicated information, clearly defining concepts and avoiding pseudoscientific terms is preferable (Morgenstern et al., 2015). However, given the character of Bio-me, and as we have not studied the perception of the campaign, it remains uncertain, how children interpret the metaphor and campaign. It is also unclear, as to whether the campaign actually informs children about biobanking and contemporary biomedical research. However, our analysis demonstrates that it does not seem to support the understanding and decision-making abilities of children in relation to what is *actually* carried out in a biobank and conducted during biobank research. Instead, it encourages them to understand biobank participation as something rather individualistic, local and material, and portrays the origin of a sample—one’s own Bio-me—as being in contradiction with the data-driven and purpose-dependent products that biobanks actually offer their users.
